# Preferencias estéticas de individuos al evaluar fotografías de perfiles faciales con diferentes patrones de crecimiento sagital y vertical. Un estudio transversal

**DOI:** 10.21142/2523-2754-1104-2023-174

**Published:** 2023-12-28

**Authors:** Gina Yanin Salinas Mendoza

**Affiliations:** 1 Carrera de Estomatología, Universidad Científica del Sur. Lima, Perú. ginasalinas900@gmail.com Universidad Científica del Sur Carrera de Estomatología Universidad Científica del Sur Lima Peru

**Keywords:** ortodoncia, perfiles faciales, tejidos blandos, orthodontics, facial profiles, soft tissues

## Abstract

**Objetivo:**

Evaluar las preferencias estéticas de individuos al observar fotografías de distintos perfiles faciales con diferente patrón de crecimiento sagital y vertical.

**Materiales y métodos: Estudio:**

transversal que evaluó 300 estudiantes de una universidad privada. Se utilizaron fotografías editadas de un hombre y una mujer. La maxila fue movida en dirección vertical, mientras que la mandíbula fue movida en dirección sagital. Se crearon varias combinaciones del perfil fotográfico de los dos individuos. Estos movimientos crearon 9 perfiles fotográficos para la mujer y el hombre. Para la confiabilidad intraobservador, se duplicaron dos perfiles de fotos de ambos sexos. Se utilizaron las pruebas de chi cuadrado y regresión logística binaria. p < 0,05.

**Resultados:**

En general, los individuos evaluados (hombres y mujeres) prefirieron el perfil de tipo 6 (65,7%), seguido por el perfil de tipo 5 (21,7%) (ligeramente convexos). Se encontró que, por cada año que aumenta un individuo, hay un 31% más posibilidades de elegir alguno de estos perfiles.

**Conclusiones:**

Ambos sexos prefieren un perfil recto o ligeramente convexo, y conforme aumenta la edad los individuos prefieren un perfil recto.

## Abstract (en)

**Objective:**

To evaluate the aesthetic preferences of individuals by observing photographs of different facial profiles with different sagittal and vertical growth patterns.

**Materials and Methods:**

Cross-sectional study that evaluated 300 students from a private university. Edited photographs of a man and a woman were used. The maxilla being moved in a vertical direction while the mandible was moved in a sagittal direction, various combinations of the photographic profile of the two individuals were created. These movements created 9 photographic profiles for the woman and the man; for intra-observer reliability, two photo profiles of both sexes were duplicated. Chi square tests and binary logistic regression were used. P<0.05.

**Results:**

In general, the evaluated individuals mostly preferred the type 6 profile (65.7%), followed by the type 5 profile (21.7%) (slightly convex). It was found that for every year of age that an individual increases, there is a 31% greater chance of choosing one of these profiles.

**Conclusions:**

Both sexes prefer a straight or slightly convex profile, and as age increases, individuals prefer a straight profile.

## INTRODUCCIÓN

La Organización Mundial de la Salud (OMS) clasifica las maloclusiones en el tercer lugar de prevalencia entre las patologías de salud bucodental ^1^^,^^2^ y cada vez más frecuente en Latinoamérica ^3^^,^^4^. Se define la maloclusión como la desarmonía del engranaje dentario donde no existe coincidencia de las piezas dentarias ^1^^,^^5^. Los huesos faciales aumentan de tamaño y volumen a medida que crecemos, y no siempre tienen un desarrollo armonioso. Algunos autores plantean que el desarrollo de los rasgos faciales, después del nacimiento, dependen específicamente de estímulos que se producen durante los primeros años de vida. La respiración, masticación y deglución adecuada influyen en una morfología y estructura facial armoniosa ^5^. Tradicionalmente, en odontología y antropología física, los maxilares pequeños se han relacionado con alteraciones como el apiñamiento dental y una relación maxilomandibular alterada ^6^^-^^9^. Es importante que el clínico estudie estos fenómenos para comprender el origen de las alteraciones en el desarrollo facial, a fin de neutralizarlos o corregirlos, y asegurar así un desarrollo armonioso del rostro y el éxito del tratamiento ortodóntico, sin que se produzca recurrencia posterior ^10^.

Las maloclusiones dentarias constituyen una anomalía muy frecuente en la clínica ortodóncica. Su alta prevalencia es la razón por la que estamos en la constante búsqueda de satisfacer las necesidades funcionales y las expectativas estéticas del paciente. Por lo tanto, el análisis facial puede ser clave en el diagnóstico de ortodoncia. Varias investigaciones han demostrado que las preferencias faciales son una respuesta a la búsqueda de la belleza del rostro humano, que es común entre los observadores, pero no necesariamente se relaciona con la edad, el género o el trasfondo cultural ^11^^-^^13^. Las características visuales específicas que contribuyen al atractivo facial incluyen las proporciones, la simetría y el tamaño de la cara.

Sarul *et al*. ^14^ describieron la importancia de las proporciones divinas y argumentó que existen muchas estructuras faciales asociadas con tejidos óseos. Arnett *et al*. ^15^ buscó características faciales en imágenes frontales y de perfil para distinguir cuándo sería preferible un tratamiento de ortodoncia u ortoquirúrgico. Ackermann y Profitt ^16^ informaron que los tejidos blandos proporcionan los límites dentro de los cuales el ortodoncista debe ajustar el tamaño del arco y la posición del maxilar-mandíbula. Así mismo, algunos autores ^15^^-^^18^ determinaron la importancia de los tejidos blandos en el tratamiento de ortodoncia y su relación directa con los tejidos duros.

La apariencia es una parte importante de la aceptación y el reconocimiento de todo ser humano. La aceptación social, el bienestar psicológico y la autoestima individual están íntimamente relacionados con ella. 

Para un ortodoncista, la belleza se vuelve práctica, ya que una de sus aspiraciones es lograr resultados funcionales y estéticos al final del tratamiento ^8^. De igual forma, para una correcta planificación del tratamiento ortodóntico, es muy importante considerar el patrón facial, ya que la aplicación de biomecánicas puede dar como resultado diferentes respuestas cuando se aplican a diferentes pacientes con patologías similares ^19^^-^^25^. 

En este sentido, el propósito del presente estudio fue evaluar las preferencias estéticas de los individuos al observar fotografías de distintos perfiles faciales con diferente patrón de crecimiento sagital y vertical, para conocer si sus preferencias son similares a las reportadas en otros estudios. 

## MATERIALES Y MÉTODOS

El estudio fue aprobado por el Comité Institucional de Ética en Investigación de la Universidad Científica del Sur, con código de registro PRE-8-2022-0046. El presente estudio fue observacional, transversal y prospectivo. La población incluyó a estudiantes de pregrado de la Universidad Científica del Sur de tres facultades, quienes fueron encuestados entre julio y agosto de 2023, y sumaron 2100 alumnos. Se realizó un estudio piloto con 60 participantes, para determinar el tamaño de la muestra del presente estudio. Se usó la fórmula para la estimación de una proporción con un nivel de confianza del 95%, una precisión del 5% y una proporción estimada del 67%, lo que dio como resultado un tamaño muestral de 293 encuestados, pero finalmente se evaluó a 300 encuestados. 

**Figura 1:**
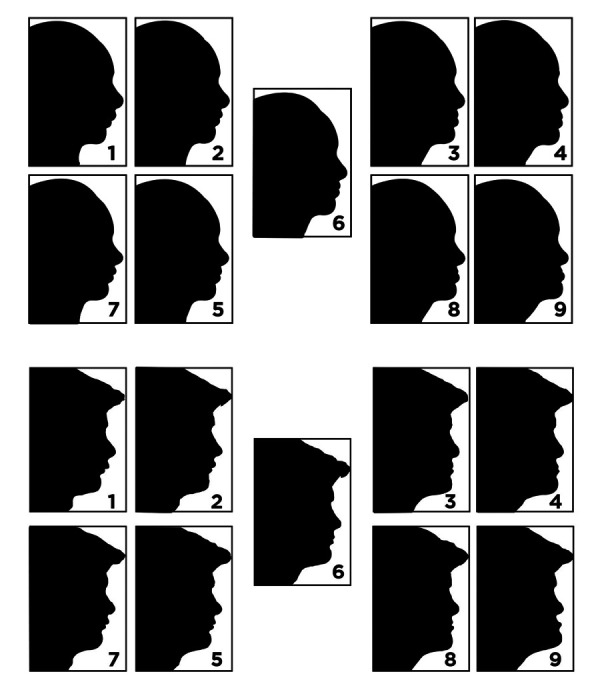
Posibilidad de elecciones del perfil facial de mujeres (parte superior, primeras dos filas) y varones (parte inferior, últimas dos filas).

Los criterios de inclusión fueron alumnos mayores de 17 años que no pertenecieran a la Facultad de Ciencias de Salud y que firmaron un consentimiento informado. Los criterios de exclusión fueron individuos con problemas mentales que pudieran dificultar la evaluación del cuestionario, individuos que trabajen o tengan contactos familiares cercanos con odontólogos en general o especialistas. Los participantes fueron elegidos mediante un muestreo no probabilístico de casos consecutivos. Para el presente estudio se utilizaron las fotografías editadas del artículo de Tugran y Baka ^26^ (Figura 1). Se editaron las fotografías de perfil de un hombre y una mujer en el programa de diseño cefalométrico Quick Ceph 2000 (Quick Ceph Systems, San Diego, Cali). La maxila fue editada en sentido vertical y movida en dirección vertical, mientras que la mandíbula fue editada en dirección sagital, para crear varias combinaciones del perfil fotográfico de ambos individuos. Los movimientos de la maxila y la mandíbula se realizaron con intervalos de 4 mm. Estos incrementos se basaron en el estudio de Romani *et al*. ^24^.

Los cambios de dirección maxilar fueron hechos con movimientos verticales de +8 mm, +4 mm, 0 mm, -4 mm y -8 mm, a fin de simular una osteotomía Lefort 1. Los cambios en la dirección mandibular se realizaron con movimientos sagitales de +8 mm, +4 mm, 0 mm, -4 mm, -8 mm, para simular la osteotomía de fisura sagital bilateral. El signo + en los movimientos verticales maxilares indica aumento del tamaño vertical y muestra prognatia en los movimientos mandibulares sagitales. Estos movimientos fueron combinados en direcciones verticales y sagitales, con el fin de crear 9 perfiles fotográficos para la mujer y el hombre. Se generaron las siguientes combinaciones: + 4 mm/+4 mm, +8 mm/+8 mm, -4 mm/-4 mm, -8 mm/-8 mm, +4mm/-4 mm, -4 mm/+4 mm, +8 mm/-8 mm, -8 mm/+8 mm y 0/0 mm. El primer valor muestra la dirección del movimiento vertical y el segundo valor muestra la dirección del movimiento sagital. Se generaron 9 perfiles fotográficos para la mujer y el hombre en blanco y negro, los cuales fueron impresos por separado en hojas A4. Todos los participantes evaluaron las fotografías de los perfiles fotográficos desde un punto de vista estético, según su sexo: hombres evalúan fotografías de hombres y mujeres evalúan fotografías de mujeres. Las fotografías fueron numeradas y los participantes marcaron el número de la imagen que más les agradó.

El análisis estadístico fue realizado con ayuda del programa SPSS para Windows, versión 19.0, y utilizó las pruebas de chi cuadrado y regresión logística binaria. Se trabajó con un nivel de significancia de p < 0,05. 

## RESULTADOS

En la Tabla 1, se observa la distribución de la muestra por sexo y edad; no se encontró diferencia significativa según edad (p = 0,392). La Tabla 2 muestra la preferencia del tipo de perfil según sexo, y no se encontró asociación entre ambas variables (p = 0,979). En general, los individuos evaluados prefirieron mayoritariamente el perfil de tipo 6 (65,7%), seguido del perfil de tipo 5 (21,7%) (Tabla 3). El análisis de regresión logística determinó que el sexo no es una variable asociada con las preferencias estéticas del tipo de perfil; sin embargo, la edad sí fue un factor que se asoció con las preferencias estéticas de los tipos de perfil; por cada incremento de un año en la edad del participante, la probabilidad de elegir el perfil tipo 6 se incrementa un 31%.

**Tabla 1:** Distribución de la muestra según sexo y promedio de edad

Sexo	n	Media	D. E.
Masculino	111	19,89	3,01
Femenino	189	19,60	2,78

p = 0,392, prueba de T de Student

**Tabla 2:** Preferencia del tipo de perfil en la muestra de individuos peruanos según sexo

Sexo	Tipo de perfil										Total	
		1	2	3	4	5	6	7	8	9			
Masculino	n	0	0	6	0	23	74	6	1	1	111
	%	0	0	5,40	0,00	20,70	66,70	5,40	0,90	0,90	100,00
Femenino	n	0	0	8	1	42	123	12	2	1	189
	%	0	0	4,20	0,50	22,20	65,10	6,30	1,10	0,50	100,00
Total	n	0	0	14	1	65	197	18	3	2	300
	%	0	0	4,70	0,30	21,70	65,70	6,00	1,00	0,70	100,00

p = 0,979

Prueba de chi cuadrado

**Tabla 3:** Preferencia del tipo de perfil en la muestra en general

Tipo de perfil	n	%
1	0	0
2	0	0
3	14	4,7
4	1	0,3
5	65	21,7
6	197	65,7
7	18	6
8	3	1
9	3	0,7
Total	300	100

**Tabla 4:** Regresión logística para evaluar la influencia del sexo y edad en la preferencia de los dos tipos de perfil más votados (5 y 6)

Variable predictora	p	Exp(B)	I. C. 95% para EXP(B)	
			Inferior	Superior
Sexo (masculino)	---	---	---	---
Sexo (femenino)	0,913	1,041	,507	2,138
Edad	0,002*	1,314	1,103	1,564
Constante	0,050	0,038		

* significativo

## DISCUSIÓN

En ortodoncia, se utilizan muchos estándares y métodos para crear y definir conceptos estéticos estandarizados; sin embargo, la subjetividad del concepto de belleza es el consenso de los autores. Las diferencias étnicas y raciales juegan un papel importante en la diversidad de preferencias estéticas. Arnett *et al.*^15^ sugieren que los contornos de los tejidos blandos son una guía básica para la colocación de los dientes; por consiguiente, para una correcta oclusión y óptima armonía facial.

En el presente estudio, se utilizaron las fotografías editadas del artículo de Tugran y Baka ^26^. Estas fueron adaptadas, además, con el objetivo de investigar el efecto del movimiento vertical de la maxila, mientras que la mandíbula fue movida en dirección sagital, y se crearon varias combinaciones del perfil fotográfico de los dos individuos. Los movimientos de la maxila y mandíbula se realizaron con intervalos de 4 mm. Estos incrementos se basaron en el estudio de Romani *et al*. ^25^.

Al examinar si el sexo se asociaba con las preferencias estéticas de los participantes sobre los tipos de perfiles, se determinó que no existía una asociación entre ambas variables. De este modo, el perfil de +4 mm en maxila y -4 mm en mandíbula, así como el perfil de 0 mm en maxila y 0 mm en mandíbula fueron los más votados por los participantes, es decir, había una preferencia por el perfil recto o ligeramente convexo, independientemente del sexo evaluado. De manera similar, Salehi *et al*. ^27^ encontraron que la relación mandibular de clase I fue el perfil más agradable, seguido por las clases II y III, respectivamente. Yin *et al*. ^28^ no encontraron diferencias significativas entre pacientes masculinos y femeninos en las preferencias de perfil facial. Torul *et al*. ^29^ hallaron que los perfiles rectos masculinos y femeninos eran los perfiles faciales más atractivos en todos los grupos, seguidos por los perfiles convexos. Los perfiles cóncavos fueron calificados como los menos atractivos; sin embargo, el estudio reveló que, en cuanto al género de los participantes, hubo diferencias significativas entre hombres y mujeres. Oliveira *et al*. ^30^, a pesar de utilizar una metodología diferente (fotografías de perfil en lugar de siluetas y cambios en la posición maxilar y mandibular), obtuvieron resultados que muestran cierta concordancia con el presente estudio, pues, en su caso, el perfil más estético en las mujeres fue una ligera birretrusión, seguida por una retrusión mandibular. En cambio, en el perfil de los hombres, lo más estético fue un perfil recto.

La variable edad influyó sobre las preferencias estéticas de los participantes. A medida que los participantes tuvieron una mayor edad, hubo una mayor preferencia por el perfil recto. Igualmente, Türkkahraman *et al*. ^31^ encontraron que los estudiantes recién graduados no notaron diferencia significativa, a diferencia de los universitarios graduados. Johnston *et al*. ^32^ hallaron que los más jóvenes fueron más críticos en la percepción estética que los adultos mayores.

Limitaciones: el presente estudio tuvo algunas limitaciones tales como no contar con participantes de otros grupos etarios (adultos mayores, adolescentes), y todos procedieron de una universidad particular, por lo que la capacidad de generalización de las conclusiones debe ser tomada con precaución.

## CONCLUSIONES

Ambos sexos prefieren un perfil recto o ligeramente convexo, y conforme aumenta la edad los individuos prefieren un perfil recto. El sexo no interfiere en la preferencia estética del perfil facial.

## Financiamiento:

Autofinanciado
